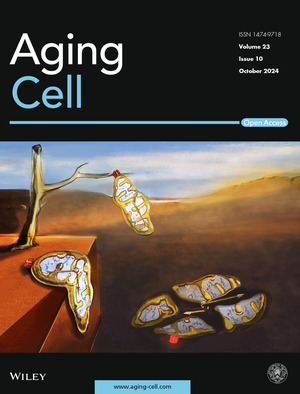# Additional Cover

**DOI:** 10.1111/acel.14379

**Published:** 2024-10-09

**Authors:** Kangkang Yu, Feifei Li, Ling Ye, Fanyuan Yu

## Abstract

Cover legend: The cover image is based on the Article *Accumulation of DNA G‐quadruplex in mitochondrial genome hallmarks mesenchymal senescence* by Kangkang Yu et al., https://doi.org/10.1111/acel.14265